# Long term effects of climate on human adaptation in the middle Gila River Valley, Arizona, America

**DOI:** 10.1007/s12685-015-0145-7

**Published:** 2015-09-03

**Authors:** Tianduowa Zhu, M. W. Ertsen, N. C. van der Giesen

**Affiliations:** Delft University of Technology, PO Box 5048, 2600 GA Delft, The Netherlands

**Keywords:** Ancient canals, Evolutionary perspective, Climate, Crop productivity

## Abstract

The Hohokam, an irrigation-based society in the American South West, used the river valleys of the Salt and Gila Rivers between 500 and 1500 AD to grow their crops. Such irrigated crops are linking human agency, water sources and the general natural environment. In order to grow crops, water available through rain and river flows needs to be diverted to land where the plants are grown. With a focus on the Gila River, this paper uses the potential harvest of maize (a main Hohokam crop) as a proxy for evaluating the influence of natural water availability and climatic changes on irrigation options for maize. Available climate variables derived from tree-ring proxies are downscaled. These downscaled data are used as input for a crop growth model for the entire sequence of Hohokam occupation along the Gila River. The results of the crop model are used to discuss the potential influence of climatic variability on Hohokam irrigation and society. The results will show that climatic change alone cannot be used as an explanation for developments in Hohokam irrigation. Societal development resulting in growing population and extensive irrigation systems increasing pressure on water sources over time would have been a key factor to include to understand Hohokam society between 500 and 1500 AD.

## Introduction

Water is and was vital for societies, and as such its manipulation has been a key for societies to emerge and prosper. The intimate linkages between human society, human agents and their water environments are expressed in ancient irrigation systems, like those of the Hohokam in the American South West discussed in this paper. In general, the evolution of ancient canals provides a distinctive insight into human adaptation under strategies in response to climate. An irrigation canal is an intentional modification of the environment, performed with awareness and attention of human beings; at the same time, irrigation systems create human activities and social relations. Ancient irrigation systems are highly suitable to study these dynamics, given their importance for societal evolution as main producers of food for the population. The variation in human population is generally assumed to be correlated with the variation in food productivity in early agrarian society, with crop yields variation linked to water availability.

In this study we focus on the process of human adaptation under climate variation in the context of Hohokam irrigation on the long term between 450 and 1450 AD. The aim of the overall project is to use this irrigation history to quantify the co-evolution of humans and nature. Human actions, climate and food production are considered as the pivot. We apply a crop growth model (CGM) to calculate the irrigation demands for the potential harvest of prehistoric maize and consider this as a proxy for evaluating how difficult it may have been to grow the crop when only considering climate variables. Human dynamics are estimated through the study of the development of canals and settlements. Climate variables are derived from the reconstruction and downscaling of tree-ring data.

The study on Hohokam food production is situated within the general debate on human niche construction (HNC), to understand human bio-social evolution (Odling-Smee et al. 2000, 2003; Laland et al. 2001; Ihara and Feldman 2004; Borenstein et al. 2006; Laland and Brown 2006; Odling-Smee 2006; Lipatov et al. 2011; Riede 2011). The basic concept of HNC is that humans not just adapt to environments but in part also construct their environments; this constructed environment on its turn influences human evolution (Lewontin et al. 1982 Laland et al. 2001). Studying long-term process with quantitative methods is a prominent feature of HNC. Some attempts have been made to discuss the evolutionary process of early irrigation canals with the theory of HNC. For example, Lansing and Fox (2011) suggest that niche construction should account for the “engineered landscape” of irrigation canals, next to biological phenomena.

Ertsen (2010) indicates that such an engineered irrigated landscape has structuring properties. Hydraulic behavior of irrigation systems resulting from human action partly enables and partly constrains new human actions. As such, we can consider the constructed canal environment as a niche with potential influence on human evolution. Humans modify the landscape and hydrological environment in the process of canals construction and the operation of these systems. The evolutionary process of canal changes in turn affects human development. Application of HNC to the evolutionary process of development of ancient canals, can help to quantify how humans respond to and adapt their direct environment in the context of climatic variation. This paper focuses on smaller-scale canal systems, localized within and closely related to naturally occurring conditions, and organized by local communities. Although there is no reason to assume that large scale imperial water systems did not construct human niches in the meaning as discussed, a focus on emergence of smaller systems at longer time scales allows further study of short term human actions within this longer time frame.

The prehistoric Hohokam canals in the middle Gila River valley of Arizona, America can be tracked back to two thousands years ago. A large amount of archeological surveys and research have been done since the 19th century (Bandelier 1892; Larson 1926; Midvale 1935, 1946, 1963, 1965, 1972; Haury 1937, 1976; Motsinger 1993; Ravesloot and Waters 2002; Woodson 2009, 2010). Hohokam canals are known along the Gila and Salt Rivers. Compared to several systems on the Salt River, the systems in the middle Gila River valley are quite small in size. Most scholars agree that an ‘irrigation community’ existed to conduct inter-village management of irrigation on the canal systems of the middle Gila area, which was separate from village administrations (Doyel 1979; Crown 1987; Hunt et al. 2005; Woodson 2010). Irrigation communities are defined as ‘autonomous, self-contained organization’ without intervention from an ‘external authority structure’ (Hunt 1988; Doyel 1991; Woodson 2010). With regards to the scale, emergence and organization, therefore, the canals systems in the middle Gila River valley are highly suitable to study HNC.

In this study on interactions between climate (change) and human dynamics, we concentrate on potential food productivity. A combination of maize, beans and cucurbita pepo (the ‘maize-bean-pumpkin complex’) has been considered the major domestic crop over prehispanic Native American agriculture (Castetter and Willis 1942; Hunt and Ingram unpublished). Beans provide nutrients for the growing needs of maize and squash; the maize stalks enable beans to climb up in turn; and the big leaves of squash at low ground keep weeds down and reduce the evaporation of soil water. Information about traditional maizes is relatively abundant, but little is known on beans and cucurbita pepo. Hence, the paper studies maize productivity under irrigation. The approach is to directly relate climatic conditions (temperature and rainfall) to possibilities for crop production over this period. A scenario on the longer term context of irrigation development in the middle Gila River valley is simulated. The temporal and spatial development of ancient canals and settlements under study are discussed, as those canals are modified, dynamically functioning at which crops are grown. The total water requirements are calculated on a basis of the potential arable. Thus we do not discuss the options to distribute the available water with the canal systems. The study emphasizes the water demands rather than the water availability for farmers within that larger area. The aim is to assess the water amount needed for irrigating all the command land and see whether changes in the water demand would have been a potential problem for the Hohokam. Crop water requirements are simulated with a CGM along the temporal scale, using regional climate data as input based on tree-ring reconstruction data, as explained below and in Ertsen et al. (2014).

## Niche construction theory, adaptation, and ancient canals

Niche construction theory (NCT) originated as a branch of evolutionary biology in the 1980s (Lewontin et al. 1982; Lewontin 1983). NCT could be fundamentally recognized as an improvement of standard evolutionary theory (SET). NCT states that evolutionary changes in both organisms and environments depend on the states of both the organisms and the environments (Lewontin et al. 1982; Lewontin 1983), as ‘an evolutionary process whereby organisms modify their environment and thereby influence their own and other species’ evolution’ (Odling-Smee et al. 2003; cited in Kendal et al. 2011, p. 785). That is to say, ‘through niche construction organisms not only influence the nature of their world, but also in part determine the selection pressures to which they and their descendants are exposed, and they do so in a non-random manner’ (Day et al. 2003).

NCT explains two processes of the interaction between organisms and environments: (1) organisms are partly adapting and partly constructing their habitat, with the consequence that (2) ‘organism-induced’ changes in environment selectively shape organisms in turn. The interaction of above two processes moves in endless ‘loops’. From this point of view, organisms adapt to and change environments as a consequence of the reciprocal and dynamic relationship between natural selection and niche construction (Laland and Brown 2006). Niche construction modifies natural selection, and natural selection determines niche construction; the combination of them contributes to adaptation of organisms (Odling-Smee et al. 2003; Laland and Sterelny 2006). Therefore, capturing the roles of NCT as an endogenous mechanism is an important way of achieving the process of adaptation, and of exploring the interaction between organisms and environments.

At present, the perspective of niche construction has been particularly explored to interpret human behavior and society as a quantitative evolutionary method; all the current theories of HNC emphasize the co-evolution of human biological and cultural aspects (Laland et al. 2001; Laland and Brown 2006; Lansing and Fox 2011; Riede 2011; Iriki and Taoka 2012). However, in the course of human evolution, the effects of NCT not only incorporate gene and culture, but also reflect on the tools and techniques used by humans, or landscapes changed by humans. Irrigation canals essentially are the products of humans adapting themselves to nature. The origin of canals might have been trial and error. When farmers found that delivering water flows by canals might increase their harvest, they were likely to extend the network of canals. The expansion of canals consequently amplified crop yields, possibly together with an increase of sediments in canals and on fields. Increase of food subsequently enlarged the human population; but at the same time, accumulative sedimentation in canals required more laborers to remove the silts. From the standpoint of HNC, food could be considered as a positive response and sediments as a negative response when humans modified their environment by means of canals. In this situation, humans themselves created both negative and positive outputs of an irrigation system. The balance of positive and negative consequences would have affected the rate or direction of the canals’ development; in such a way, humans’ adaptation was altered evolutionally.

**Fig. 1:**
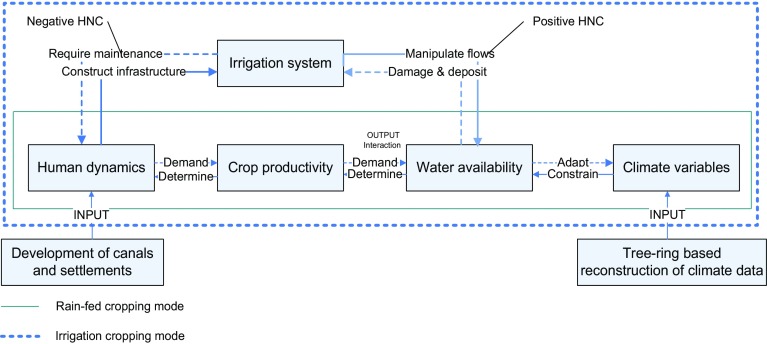
Framework of understanding the link between humans and climate

Figure 1 shows that, humans interact with the weather (on larger temporal and spatial scale society interacts with climate), which is expressed in crop and water relations in both rain-fed and irrigation crop production. The human population depends on crop productivity that is determined by water availability; the climate and weather constrain water availability, thereby having impacts on human dynamics. In attempting to adapt to a dry environment and improve moisture conditions, humans build canals to deliver water to the farmland, thus constructing the so-called irrigation cropping mode in the framework. During the process of water manipulation, irrigation systems are able to stabilize water supply for agriculture: positive HNC. Meanwhile, flooding damage and sedimentation also bring about a negative influence to humans. A major link between human dynamics and climate variables in the context of irrigation is the interaction between food productivity and water availability. Modeling crop production and water demand on irrigation over time, allows us to explore the co-evolutional process of humans and climate.

## Irrigation and settlement

**Fig. 2:**
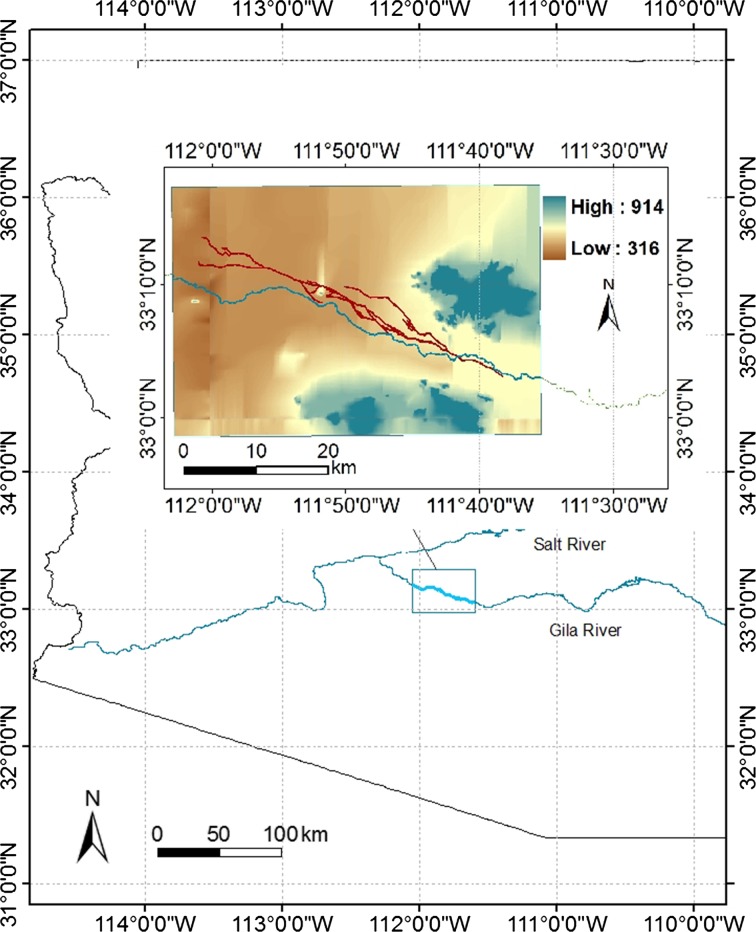
Map of Study area

The area of interest is located in the middle Gila River valley, south-central Arizona, United States of America, and encompasses four small-scale canal systems named Granite Knob, Santan, Gila Butte, and Snaketown respectively from upstream to downstream (Fig. 2). The climate of the middle Gila River Valley is hot and dry. According to the observations at Chandler Height station (1954–2000, NOAA) the mean annual minimum temperature is 13 °C, with a maximum average value in July of 24.5 °C, and a minimum average value in January of 3.7 °C. The mean annual maximum temperature is 29.4 °C, with the July maximum value averaging 40 °C and the January minimum value averaging 18 °C. The mean annual precipitation is relatively low, varying between 100 mm and 400 mm per year. The driest months are April, May and June; rainfall is largely concentrated in July, August, December and January, presenting a bimodal distribution (1896–1987, Climatic Division 6, NOAA). In spite of the Pacific frontal storms responsible for the precipitation of winter and spring, summer monsoons dominate precipitation in the area (Masse 1991). Monsoon rainfalls randomly occur at very small spatial scale for a short time, but could provide a fair amount of water locally (Fish and Nabhan 1991, p. 35). However, before a more detailed analysis of this aspect is made, the potential benefit or harm of the monsoon rain remains something of an unknown field of knowledge.

The altitudes of the study region range from around 300 m above sea level in the northwest to about 950 m in the southeast, with 335 m at the Gila River near Pima Butte, 457 m on the top of Gila Butte, and over 945 m in the Santan Mountains (Woodson 2010). The area is characterized by low-gradient desert plains and low-lying mountain upland (Huckleberry 1993). The topography of the alluvial plains along the middle Gila River enables the construction of gravity controlled irrigation canals. Agriculture might originate in 300 AD in the Gila River basin, although evidence of earlier agriculture elsewhere in the larger region has been found (Ertsen et al. 2014). It is generally accepted that the main cultivated crops were maize, bean, squash and cotton along the whole Gila River (Bohrer 1970; Haury 1976; Gasser and Kwiatkowski 1991). The limited precipitation is incapable of supporting the water needs for full growth of these crops under natural conditions. Thus, agriculture is assumed to be dominantly dependent on irrigation. The initial canals are likely to have occurred as early as the appearance of crops. Irrigation being a necessity for yielding adequate food for humans’ survival must have been a critical component in the behavioral responses to environmental variation in the Gila area.

The area is conventionally known as a part of the Hohokam, as defined by its culture. Hohokam is notable for its irrigated agriculture, which has received considerable attention from different fields (Haury 1976; Graybill 1989; Howard 1993; Hunt et al. 2005). The Hohokam society has been overwhelmingly considered as a prehistoric agricultural society, achieving a remarkable adaption to their arid environment in the North American Southwest (Haury 1976; Doyel 1979; Bayman 2001). Reconstructions of canals and settlements in the study area has been substantially investigated in previous research (Bandelier 1892; Southworth 1914, 1915; Larson 1926; Midvale 1935, 1946, 1963, 1965, 1972; Haury 1937, 1976; Gladwin 1948; Woodbury 1976; Schiffer 1982; Dean 1991; Motsinger 1993; Gregory and Douglas 1994; Neily et al. 1999, 2000; Ravesloot and Waters 2002; Woodson 2006, 2009, 2010; Loendorf et al. 2007; Ravesloot 2007; Burt 2007; Fertelmes and Loendorf 2010). In this study, the recent Hohokam chronology in the Gila area completed by Woodson (2010) is followed.

**Fig. 3:**
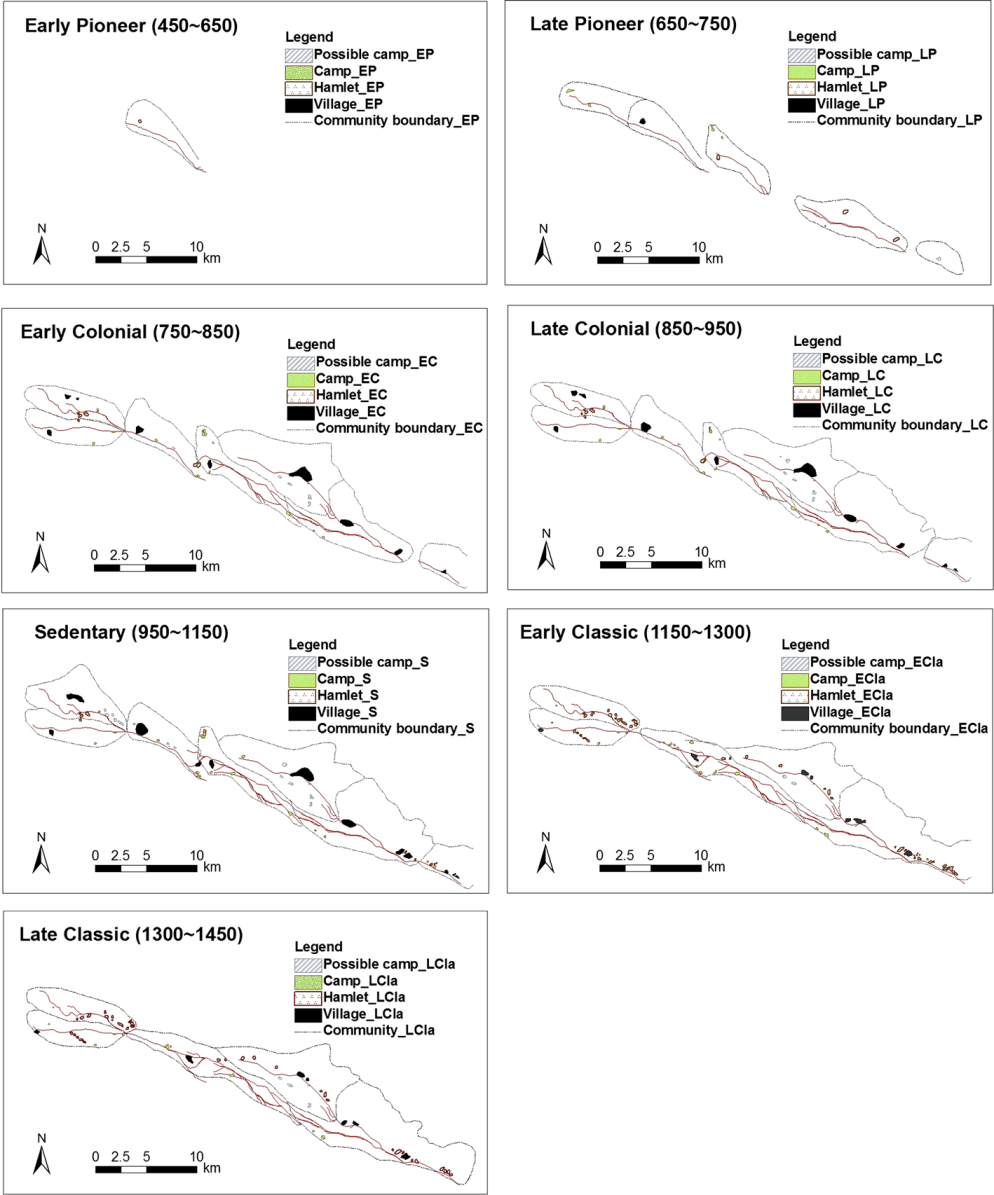
Maps of canals and settlements development (extracted from Woodson 2010)

Hohokam culture is classified into four periods: Pioneer, Colonial, Sedentary and Classic Periods, and each of them are subdivided into the Early and Late periods except of the Sedentary (for dates see below). Therefore, the time period in the evolution timeframe of the area contains Early Pioneer, Late Pioneer, Early Colonial, Late Colonial, Sedentary, Early Classic, and Late Classic periods. The maps of canals and settlements development through time are shown in Fig. 3. The development of the canals and settlements is evaluated in the chronological order of the above-mentioned time classification. With respect to the habitation types, settlements are defined as village, hamlet, and camp as defined by the size (Gregory and Douglas 1994; cited in Woodson 2010). A village represents a residence with a population of more than 100 people, occupied over a relatively long period; a hamlet is a relatively smaller site with an occupation on a year-round basis; a camp is a short-stay and family-size settlement.

The first canal in the Gila area appears to have been built in Snaketown during the Early Pioneer period (450–650 AD); its habitation would have been at hamlet-level. No settlements in the Gila Butte, Santan and Granite Knob were found yet. In the Late Pioneer period (650–750 AD), the existing canal in Snaketown was doubled in length to the west, and the previous hamlet had extended to a village-level settlement. One camp (with two more possible) appeared next to the new canal of Snaketown. Southeast of Snaketown, the Gila Butte canal was established as well. Located at the tail end of the canal, a hamlet called ‘Gila Butte Site’ was likely to be built simultaneously to the large settlements. Woodson (2010) assumes that the residents of the Gila Butte Site were largely focused on their own canal system for subsistence purposes rather than the Snaketown Canal System. In addition, two camps emerged north of Gila Butte and a third camp was probably established at the southwestern foot of Gila Butte. Southeast of Gila Butte, the first canal in the Santan was constructed; two hamlet-size settlements were occupied at the north of the Santan canal (see more details in Woodson 2010).

During the Early Colonial period (750–850 AD), the entire canal system was enlarged three times and almost reached its maximum extent. In Snaketown, a new extension to the west was constructed based on the canal of the Late Pioneer period, and a south branch canal was built too. Three primary villages existed at this time. The two possible camps in the Late Pioneer period had expanded to a village still with two recognizable settlements. A new village came into being along the new south branch canal. There were four hamlets in the south of the North branch of the Snaketown canal. Six new camps and one possible camp were established as well. In Gila Butte, some small canals were built to the west, and the earlier hamlet grew into a village. The Santan canal system was extended to connect with Gila butte. At the north of Santan, another canal was constructed almost as long as the south one. It had three villages, and several (possible) camps. Granite Knob only contained one canal and one small village at the time. During the Late Colonial period (850–950 AD), the configuration of canal systems and settlements had changed very little compared with the Early Colonial period. The three villages of Snaketown slightly grew in size and a small village was added at Granite Knob.

In the Sedentary period (950–1150 AD), there was not much new construction on the canal systems and human settlement reached a peak. In Snaketown, three villages had increased in size, and a previously occupied hamlet near to Gila Butte had grown to village size. Eight new possible camps might have occurred besides the six existing camps. The upper part of the village at Santan expanded in intensity of use as well. The settlements in Granite Knob included a dual village as the one in the Snaketown and added a hamlet. During the Early Classic period (1150–1300 AD), the settlement sizes declined. A new canal was built between the Gila Butte Canal and the Snaketown Canal. In spite of one new small village appearing at the far western side of the Santan south branch canal, two previous villages had been abandoned. The habitants redistributed themselves into a growing number of small-size settlements. Instead of more intensive occupation in villages, eighteen hamlets (four hamlet clusters) and thirteen camps dispersed along the Snaketown canals. The settlements in Gila Butte shrank much in size. The situation in Santan was similar to that in the Snaketown. The three big villages were replaced with three small dispersed villages and an amount of hamlets. The camps reduced by nearly half in number. The villages in Granite Knob regressed to hamlet-level. By the Late Classic period (1300–1450 AD), more settlements were abandoned.

## Climate variables

In order to explore the co-evolutional process of humans and climate, maize production and water demand on irrigation from 450 to 1450 AD are modeled. However, the challenge of doing so is to determine the variability of temperature and moisture within that timeframe first. To obtain a 1000 year record of climatic variability, tree-ring reconstructed data (Species: Pinus edulis, Pinus ponderosa, Pseudotsuga menziesii, and Pinus aristata, (Salzer and Kipfmueller 2005)) with a millennium span are used in this study. The climate reconstruction based on tree-ring proxy records has been considerably utilized with respect to its relatively high resolution and reliability (Kohler 2012). In the selection of potential tree-ring indexed series, four principles were considered:

1. the tree-ring sites should be relatively close to the study area;
2. the tree-ring should be distinctively sensitive to climatic variables (temperature and precipitation);
3. the dataset should cover the main period one is interested in (which would be 450–1450 AD for the main Hohokam period);
4. and the data set should correlate with the study area.

There are two main issues to deal with before one can model maize production. The altitudes and climatic zones of the tree sites and the study area are different, with tree-ring sites located at higher altitudes with lower temperatures and relatively substantial rainfall. Furthermore, the data derived from tree-ring chronologies are at annual-scale (October–July), but more detailed (monthly or even daily) data are required to compute maize productivity as maize is a seasonal crop. Hence, it is necessary to downscale the yearly data into month-scale data. Details of the method we used can be found in Ertsen et al. (2014); in this paper, these results are summarized to simulate maize productivity.

**Fig. 4:**
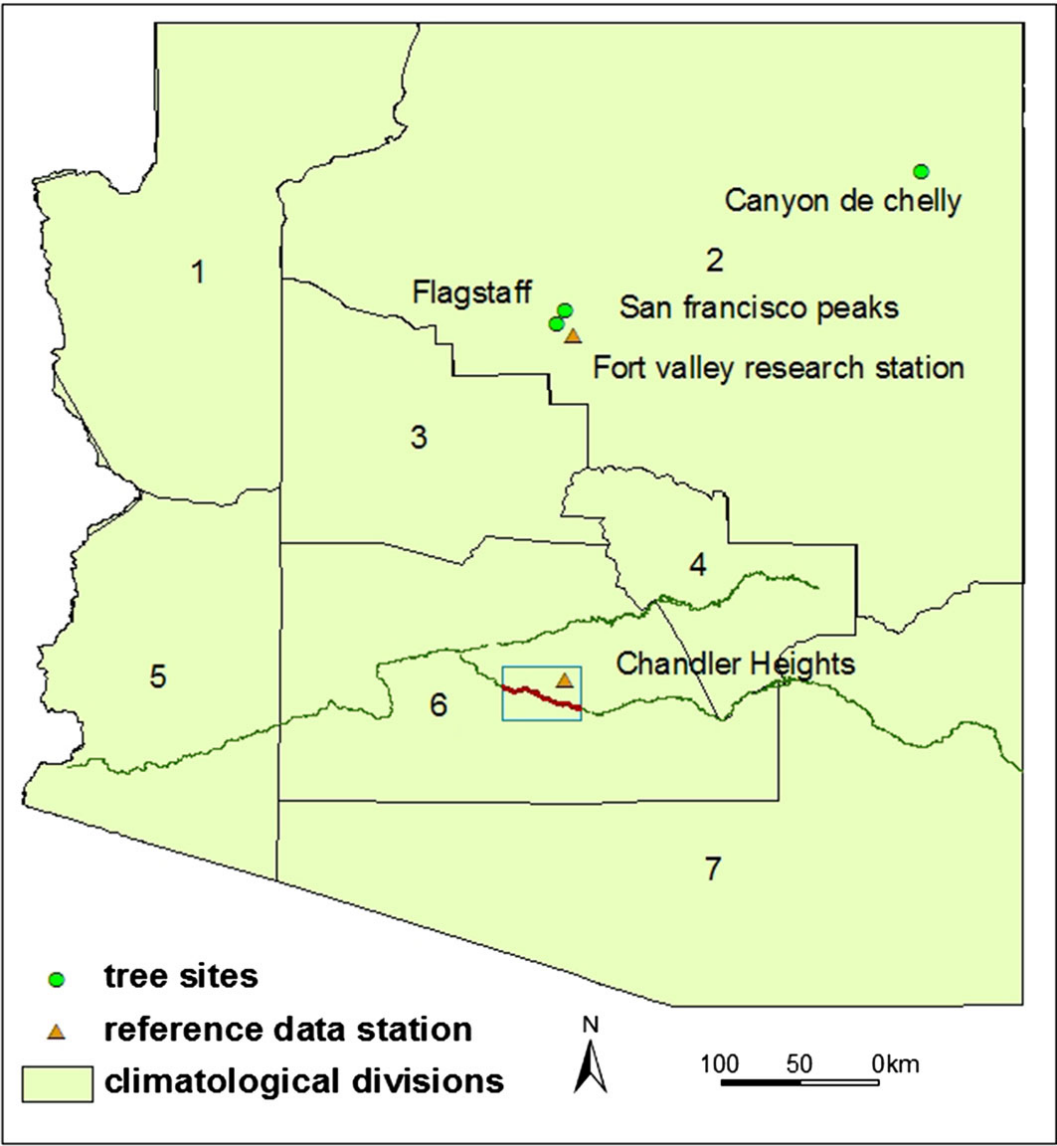
Location of tree-sites

Reconstructed data (https://www.ncdc.noaa.gov/paleo/pubs/salzer2005/salzer2005.html) from Salzer and Kipfmueller (2005) was selected in this study, which cover annual precipitation (in October–July) for a 1425 year period (570–1994 AD) and annual mean-maximum temperature for a 2262 year period (250 BC-1997 AD), based on calibrated precipitation series with data from NOAA climate division 2 (CD2) and temperature series with data from Fort Valley research station. There are three tree-ring chronologies in the lower forest border of Arizona (Flagstaff, Canyon de Chelly, and Navajo Mountain) being used for precipitation reconstruction and one in San Francisco Peaks in Northern Arizona (Flagstaff) for temperature reconstruction, as shown in Fig. 4. It is wise to assume in general that climatic variability has not changed over time, to be able to use observed data in the analysis. The tree-ring data series shows no evidence of climate changes in the uplands. We assumed that the monthly observed data in the lowlands and the difference/ratio between yearly reconstructed tree-ring data and yearly observed data in the uplands could be related.

The original tree sites and Hohokam irrigated area are not on the same altitude. All tree-ring sites are located above 2000 meters, but the Hohokam area is located around 450 meter, both above sea level. This also yields different climatic zones: tree-ring sites are associated with the CD2 zone, with low temperatures and relatively substantial rainfall, whereas the Hohokam main area is within the hot and dry climate of the CD6 zone. Correlation between temperature and rainfall were applied to the two zones. The resulting correlation coefficient r for temperature was 0.99, somewhat higher than the coefficient for precipitation of 0.83. This means that rainfall and temperature in study area were strongly correlated with the same in the tree site zone.

In order to use climatic data on a meaningful scale to simulate crop productivity and water use, we downscaled the annual data into month-scale ones using the change factor (CF) method (Chen et al. 2011), which adjusts observed time series by adding the difference (for temperature) or multiplying the ratio (for precipitation) between future and present climates. One of the advantages of the CF method is its straightforward application. For application, the equations were modified slightly by linking the change factors between reconstructed tree-ring data and upland observed data. As such, the CF method could take into account climatic differences between high and low altitudes, by linking climate variability at high altitudes to the same in the lowland, along the equations below. The simulated series of precipitation were validated by comparing month-distribution percentage with the observations for the same percentage. As discussed in Ertsen et al. (2014), the model captured the precipitation frequency at monthly scale well.

## Crop yields

With climatic data available for simulating maize growth, it is time to turn to the maize crop itself. Estimations on the yields of traditional maize have been widely conducted in or nearby the study areas (King 1923, King and Leding 1926, King and Loomis 1932, cited in Hunt and Ingram unpublished 2; Castetter and Willis 1942; Burns 1983; Petersen 1987; Van West 1994; Diehl 1997, 2005; Muenchrath et al. 2002; Hunt and Ingram unpublished 2; Kohler 2012; Benson et al. 2013). A literature review was given with respect to three aspects: location, period, and methodology, see Table 1. With regard to location, the available studies can be classified into high altitude areas (Mesa County, Southwestern Colorado), and lowlands (Pima-Papago, Sacaton and Middle Gila). With respect to the period of study period of maize yields, previous work includes modern times (early 20th century), and prehistoric periods. However, the existing models of prehistoric maize productivity fundamentally depend on data sets collected from modern field observations. In terms of methodology, there are four main groups: ethnographic case studies, energetic cost estimates, experimental research, and modeling simulations. In addition to the perspectives highlighted above, the specific maize types also differ in prior studies. Some scholars have even suggested that it is problematic to shift prehistoric maize specimens to modern races of maize (Diehl 2005).

**Table 1:** Previous studies on traditional maize yields

Source	Location	Periods	Methodology	Condition	Yields (kg/ha)
Castetter and Willis (1942)	Pima-Papago	Before 1940s	Ethnographic reports	Native condition	628–753
Diehl (1997), Diehl (2005)	Tucson	Prehistoric	Energetic cost-estimates	/	300
King 1923, King and Leding 1926, King and Loomis 1932; cited in Hunt and Ingram, unpublished	Sacaton Lowlands	1920s	Experiment	Irrigation	1900
Burns (1983)	Southwestern Colorado	1931–1960	Experiment	/	251–1255
Muenchrath et al (2002)	Mesa county 1838–2434 m	1998	Field observation	Runoff/floodwater	0–1841
Hunt and Ingram unpublished 2	Middle Gila	1912–1929	Simulation	Irrigation	551–974 season 1; 971–1777 season 2
Kohler (2012)	Southwestern Colorado	600–1300	Simulation	/	Based on Burns (1983)
Benson et al. (2013)	Southwestern Colorado	480–2011	Simulation	/	Based on Burns (1983)
This paper	Middle Gila	570–1450	Simulation	Full irrigation impact of moisture	7600 (Potential)

Castetter and Willis (1942) performed an ethnographic study on traditional maize cultivation at Pima and Papago. Their study covered planting calendars, crop characteristics, production, and consumption. From the standpoint of energetic consumption, Diehl (1997, 2005) estimated the yield of early maize under the hypothesis of optimal energetic return rates for foragers. Several experimental studies are available on productivity of traditional maizes (several publications of King, Leding, and Loomis (1920s–1930s), Burns (1983), and Muenchrath et al. (2002)). Finally, simulation models based on experimental data have been used in Hunt and Ingram (unpublished); Kohler (2012), and Benson et al. (2013) to estimate maize productivity. Kohler (2012) normalized maize yields from 1931 to 1960 and applied tree-ring based reconstructions of temperature to estimate the palmer drought severity index (PDSI) to produce prehistoric maize yields. Benson et al. (2013) considers that Kohler’s model attempts to involve ‘all processes impacting maize productivity’, which makes some of the model’s parameterizations problematic. In the paper, a simpler modeling approach to estimating maize yields is presented, by estimating maize production as a function of soil organic-nitrogen concentrations and water-year precipitation (which is also based on tree-ring reconstructed data).

With regard to perceiving the variance of prehistoric maize yields in the long term, along with tree-ring reconstructed data is a relatively precise and feasible option as mentioned in paragraph 4. The standard crop water requirement model AquaCrop of the Food and Agricultural Organization (FAO) was used in this study. We focus on the impacts of climate on maize productivity by computing maize production under the assumption of full water supply. Thus, we calculate the potential yield. What is actually harvested cannot be computed. Modeling ancient crop yields for ancient agriculture is particularly different from the simulation of modern crops. For ancient crops, it is unrealistic to obtain actual yields.

Reconstructing maize production over 1000 year period has its complexities. Again, the spatial difference between the study area (lowland) and the tree-ring sites (high altitude areas) as well as the divergence of temporal scales between the tree-ring chronology (annual) and the requirements of the model (monthly) are issues to deal with. In addition, there are several key factors determining maize productivity, such as seed variety, climate, soil environment, farmer’s behavior, insects etc. The study restricts its focus to the impact of climate variables on maize productivity. The objective of this restriction is to assess the possible role of climate in structuring human adaptation. Furthermore, no other single factor except precipitation and temperature can be reconstructed for a series of one thousand years in ancient periods. Due to these reasons, the models that rely on field observations (Kohler 2012; Benson et al. 2013) are not suitable for the study, despite those being able to estimate the variance of maize productivity at a thousand year scale. Instead, a CGM is used for calculating the irrigation demands for the potential harvest of prehistoric maize. The CGM can describe the growth process of the maize under given prehistorical environment, which incorporates four input variables: climate, soil, crop and management. With the irrigation demands of the maize in the study area, it can be estimated how difficult it may have been to grow the crop when only considering climate variables. The higher the water demand for maize, the more difficult it would have been to grow the crop. That will provide a reasonable baseline analog for how climate changes may have influenced maize growth.

Clarifying the distinction between potential yields, available yields, actual yields and consumptive yields is pivotal to understanding the modeling setup. There are several definitions available to distinguish between the four categories. Gaines and Gaines (2000) define potential yield as “the yield which is influenced by climatic and edaphic effects and is treated as a per unit variable”, and realized yield (actual yield) is “the food value obtained from the harvest”. De Wit (1958, cited in Rockstrom and Falkenmark 2000, p. 321) stresses the hydrological perspective: “for a given crop and hydro-climate, the potential yield (assuming no deficiencies at all) will be determined by climatic factors (basically incoming radiation and temperature)”. In this explanation, the climate is placed in a vital position for potential yields. The difference between potential, available and consumptive yields is explained by Schroeder (2001), who points out that “the amount of the potential yield that is harvested is referred to as available yield; … the proportion of the available yield that is consumed by humans is referred to as consumptive yield.” Not only did Schroeder explain the relationship among potential, available and consumptive yields, but also he identified available yields as actual yields.

Potential yields are assumed as a distribution of available maize under possible circumstances of climate and soil. In developing a baseline to reveal moisture effects on crop growth process, the irrigation requirements are modeled based on an acceptable range of potential yields. We have set this range starting with ideas on potential consumptive yields. In the work of Castetter and Willis (1942), consumptive yields were calculated assuming that a family with 5 people in average cultivated 0.25–2 acre (0.10–0.81 ha) of land, and the corn yield per unit ha was 12 bushels/acre (753 kg/ha). One family thus consumed 0.6–4.8 bushel corn per year. We reversed this method to relate potential yields and consumptive yields. The basal metabolic rate is about 1300–1500 kcal/day for an adult female and 1600–1800 kcal/day for an adult male (Purves and David 2004). For farmers with their high intensity of labor, the energetic costs of normal life are at least 2–3 times that of the basal metabolic rate (Diehl 1997).

Taking a 5-person family with an average 2400 kcal/day per person, the whole family would consume 4380,000 kcal per year. Castetter and Willis (1942) stated that only about 25 % of the calories came from maize within an Akimel O’odham diet. If taking this percentage for the middle Gila, there would be 1095,000 kcal of energy deriving from maize. According to Foundation of Anasazi Culture (Reed 2000), one kilogram of traditional corn could support 3476 kcal of energy. That would be 315 kg of maize needed for consumption. Given 0.1–0.81 ha of arable land for each family (half for maize, half for beans and pepo) and two cropping seasons per year (Hunt and Ingram 2007), yields should be within the range of 389–3150 kg/ha to support the basic consumption of a family. Taking the most conservative number, one hectare of farmland would have to produce 389 kg of actual maize to keep the balance between demand and supply.

This 389 kg is the actual yield. To determine potential yields from this baseline figure, all kinds of possible factors resulting in this actual yield from a given potential yield should be considered. When growing and processing maize, typical losses that can occur result from soil salinity, insects, and other factors like seed varieties, agricultural technology, and human actions (even stealing!). The paper assumes that each of these factors has a 40 % loss rate on average (based on Edwards et al. 1988; Oberle and Keeney 1990; Ismail et al. 1994; Kaufmann and Snell 1997; Gianessi and Janet 1999). Furthermore, a threshing rate of 20 % is added. All this leads to potential model yields of around 7.6 t/ha per season in the simulation. Referring to this amount of maize production, we have adjusted the Aquacrop model parameters to calculate more realistic irrigation demands for prehistoric periods. The precipitation and temperature input is the downscaled tree-ring data discussed above. The reference evaporation (ET_0_) data was calculated with the T-based Penman–Monteith equation.

Hohokam cropping intensity was likely to be twice per year with a growing cycle of 120 days per crop; the most likely cropping seasons were middle or early March to middle July and late July to middle November respectively (Hunt and Ingram 2007). At the beginning of each planting season, certain moisture and a minimum temperature are required for germination. The soil at the study site involves multiple types in the upper layer in terms of soil texture (Web soil survey, USDA). Although the soil variability does influence water demand for agriculture, the study emphasizes the impact of climate variability on crops. Therefore, the soil profile is assumed to be homogeneous as sandy-loam in the model. In future work, we will focus on the diversities of soil properties and the potential influence on accuracy and reliability of results. In addition, bunds and mulches are not applied in the model.

## Results

**Fig. 5:**
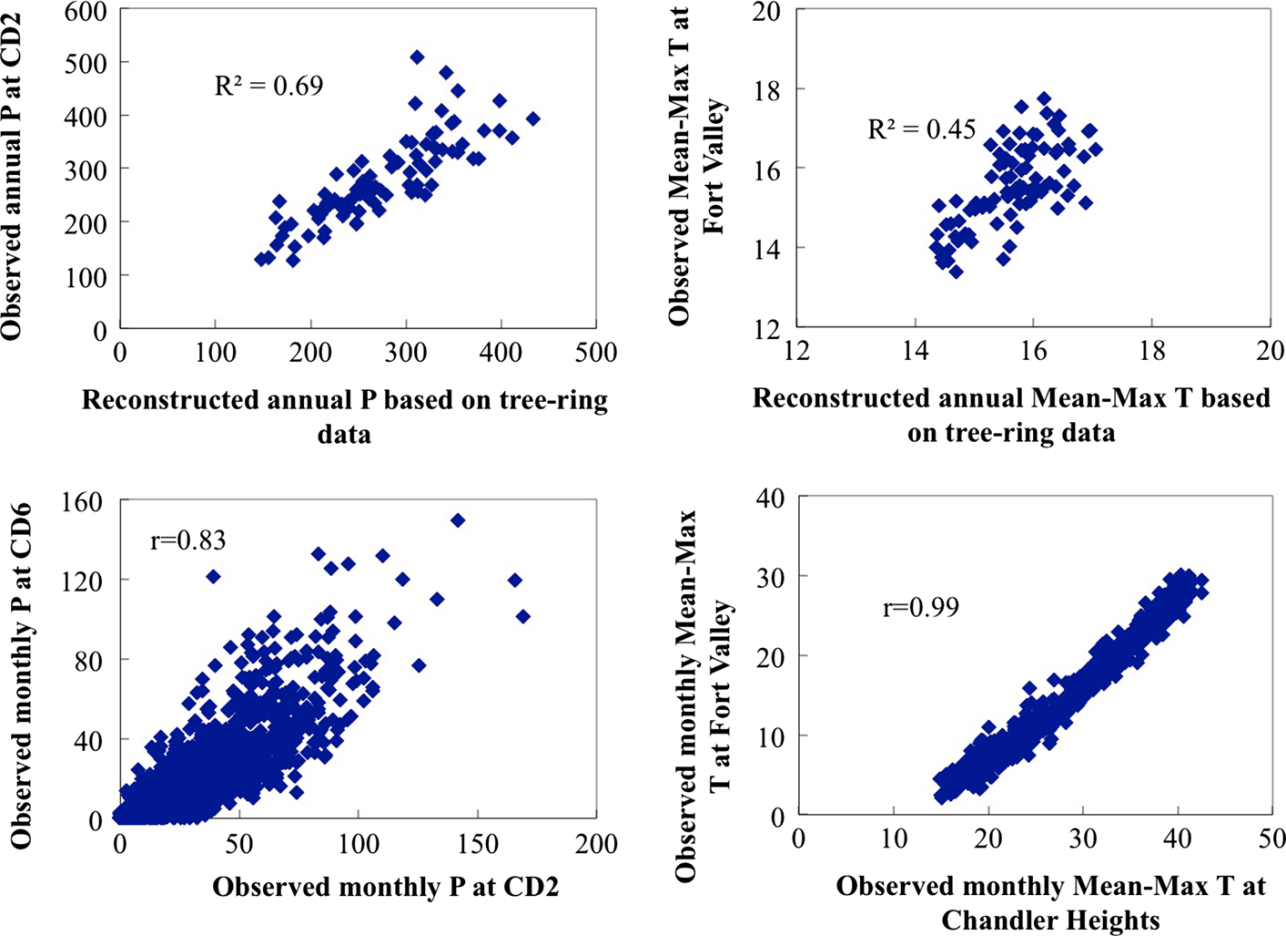
Simulated net irrigation demands and identified with extreme climatic intervals over all periods

For the reference maize yields of around 7.6 t/ha, net irrigation demands for maize were simulated for two growing seasons for a thousand year period (570–1450 AD), see Fig. 5. The results indicate that the chronology of irrigation demand basically captures the variability of rainfall on annual scale. The variation in irrigation requirements is comparable to normal fluctuations over all four Hohokam chronological periods. The results do show a difference in water needs within a year. Water demands in the first growing season are between 450 and 650 mm, while the amounts on the second mostly range from 350 to 450 mm. The difference is basically caused by the rainfall and evapotranspiration pattern. Precipitation largely takes place in July, August, December and January. The cropping seasons are assumed to be March-to-July and July-to-November respectively. As a result, crops in the second season receive more moisture than those in the first sub-season without considering irrigation. In addition, the highest ET_0_ are found in the May–July period. Therefore, the first cropping season should consume more irrigation water than the second one.

The figure shows that the net irrigation demands (unit in mm) have a good match with the so-called anomaly index (Salzer and Kipfmueller 2005). The anomaly index identifies the extreme climatic events at decadal scale, which is the most reliable scale from tree-ring data reconstruction. A positive value of the anomaly index is interpreted as wet conditions, and negative as dry conditions. In wetter periods, less water is required for maize in at least one of two seasons each year compared to other periods. During droughts, both seasons broadly demand larger quantities of water. In addition, there seem to be more droughts as well as floods during the Sedentary period compared to other periods on average. The results of first season irritation demands suggest that the probability of a demand above 600 mm during the Sedentary is higher than that during other periods, but the same is not apparent for a probability of below 500 mm. This may suggest that simulated results perform better in extreme dry events compared to extreme wet ones.

**Fig. 6:**
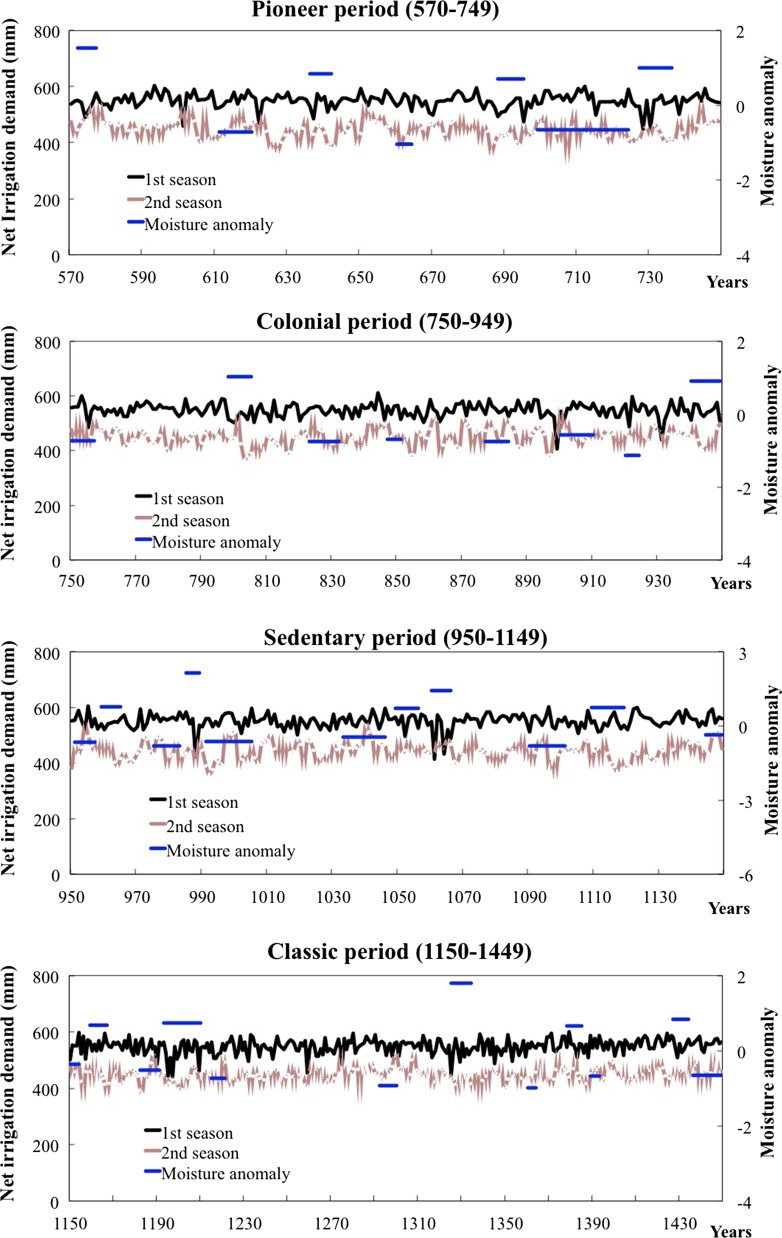

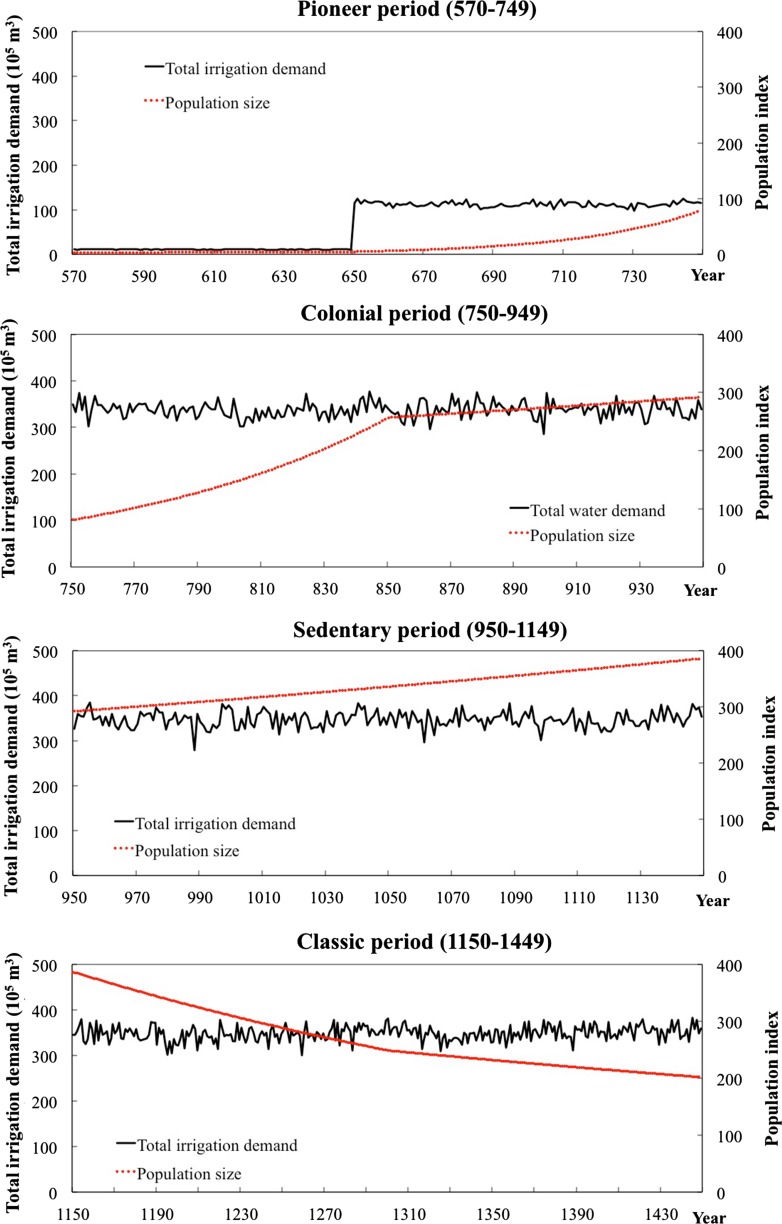
Simulated total water demands and relative population sizes over all periods

The total water requirements using the potential arable area capable of being reached by the canals are computed. The study emphasizes water demands rather than the availability of water flows. Calculating potential water demands needed for irrigating all the command land without considering water availability shows the potential stress on the system in the different periods. The command land areas, together with relative population sizes, are derived from forgoing maps of canals and settlements development; and they are shown in discrete values over temporal scale. The results of total water requirements (volume as discrete value) and relative population sizes (no dimension) are presented in Fig. 6.

The results reveal that there are two leaps in water demands in the chronology of Hohokam society along the Gila River. The first one is between the Early Pioneer and Late Pioneer periods; a later one occurs between the Late Pioneer and Early Colonial periods. The reason of this leaping is obviously because of discrete values in command land areas and associated water volumes. These transitions—which would have been more gradual in reality—are very crucial periods in HNC. However, the actual evolutionary path in these transit periods has many possibilities. Did land expansion follow population growth or was it the other way around? Figure 6 shows that the population sustains a relatively stable increase before the Classic period and reaches its peak at the end of the Sedentary period. The rate of population growth is relatively high during the Early Colonial period compared to those in the Pioneer and Sedentary periods. Contrary to other periods, the Classic period shows a decline in population.

In the simulation, potential maize yields are demonstrated to be in a similar range per hectare. Over the span of one thousand years, maize water requirements did not become a limiting factor. Changing climate conditions were not determining maize potential productivity. Total water demands are directly proportional to potential maize yields for human consumption in each period and as such an indicator of stress for society to reach a certain production. Therefore, relating water requirements and population size indicates the relationship between potential food productivity and population change, or between potential supply of and demand for food. Figure 6 reflects an upward trend in both water demand and population during the Pioneer and Colonial periods. In the Sedentary period, populations keep increasing, but water demands maintain a stable range. By the Classic period, water demands continue to be stable, but population regresses. In this respect, the period between the Sedentary and Classic periods is a crucial process of HNC as well. Some changes seem to have broken the (delicate) balance between positive and negative influences of niche construction in this period. What these changes were exactly needs further study.

## Discussion and conclusion

In this study on interactions between climate (change) and human dynamics, we have studied the relation between potential food productivity and population growth. We assessed the water amount needed for irrigating all the command area to see whether changes in the water demand would have been a potential problem for the Hohokam. We considered water needs for a reference maize harvest as a baseline for evaluating how difficult it may have been to grow the crop when only considering climate variables, providing a reasonable baseline analog for how climate changes may have influenced maize growth. The analysis has shown the occurrence of two leaps in relative water demands, one between Early Pioneer and Late Pioneer, and one between Late Pioneer and Early Colonial indicating that these were crucial transitions in HNC. The image of ‘leaping’ is artificial because of the use of discrete values in command land areas, and there are many possibilities to explain the exact shapes of these transit periods. Detailed study is needed to explain the transiting process further. In addition, the transition from Sedentary to Classic appears to be another significant process of HNC. The balance between positive and negative influences of niche construction is likely to be broken in this period.

The variation of irrigation requirements is matched with the normal fluctuation of climate over all four periods. As mentioned before, irrigation demands do not vary extremely over the modeling period; therefore, we assume that the instability of the canal systems originates from internal factors. The expansion of the canal system may increase its vulnerability and extreme climatic events—which can be found in each period—could have triggered the regression, even abandonment of the canal (Pande and Ertsen 2014).

The foregoing analysis illustrates the feasibility of tree-ring-based data in long-term modeling, taking into account traditional maize yields, using a CGM to calculate irrigation demands for the potential harvest of prehistoric maize. The concepts of potential, available, actual and consumptive yields needed to be taken into account. The irrigation requirements were modeled based on an acceptable range of potential yields, taking into account consumptive yields. The seasonal production of maize needed to be considered as well. Water demands in the first growing season appeared to be higher than that in the second because of the rainfall-evapotranspiration pattern. The simulated irrigation demands showed a relative good match with extreme climatic events. However, the model seemed to have better performance in extreme dry events than in extreme wet ones. One explanation may be that the tree-ring data are more sensitive to droughts than wet periods.

A next step would be to focus on the transformations between the phases as indicated. The actual construction and use of irrigation systems in response to changing conditions will be a main focus. Humans modify the landscape and hydrological environment in the process of canals construction. The evolutionary process of canal changes in turn affects human development. These canal systems do not work on rainfall, but on river flows. Thus, a next challenge is to build a 1000 year record of flows in the Gila River on a sufficiently low time step to be used as boundary conditions for flows in the canals. Once we have included stream flow data in the middle Gila River and how these flows were distributed between and within irrigation systems, we could determine ranges for available yields. This would possibly yield more detailed understanding of the variation of food availability and as such population densities in study area.
